# Embryonal rhabdomyosarcoma of the cervix presenting as a cervical polyp in a 16-year-old adolescent: a case report

**DOI:** 10.1186/1752-1947-8-241

**Published:** 2014-07-01

**Authors:** Sofia Jayi, Hakima Bouguern, Fatima Zohra Fdili, Hikmat Chaara, Leila Chbani, Imane Hafidi, Imane Kamaoui, Samia Arifi, Naoufal Mellas, Touria Bouhafa, Khalid Hassouni, Siham Tizniti, Afaf Laamarti, My Abdelilah Melhouf

**Affiliations:** 1 Department of Gynecology and Obstetrics, University Hospital of Fez, Fez, Morocco; 2 Department of Anatomopathology, University Hospital of Fez, Fez, Morocco; 3 Department of Radiology, University Hospital of Fez, Fez, Morocco; 4 Department of Oncology, University Hospital of Fez, Fez, Morocco; 5 Department of Radiotherapy, University Hospital of Fez, Fez, Morocco; 6 Sidi Mohammed Ben Abdellah University, Fez, Morocco; 7 37-39, Lotissement asmae, Route ain chqef, Fez, Morocco

**Keywords:** Cervix, Embryonal rhabdomyosarcoma, Management, Prognostic factors

## Abstract

**Introduction:**

Embryonal rhabdomyosarcoma of the female genital tract is rare in the cervix. It has been mainly discussed in the context of individual case studies. It tends to occur in children and young women. Treatment ranges from radical surgery to conservative surgery, followed by chemotherapy.

**Case presentation:**

A 16-year-old Moroccan adolescent girl presented to our center with a protruding mass from her vaginal introitus, as a polyp of 6cm. An examination revealed a polyp within her vagina, thought to be arising from her cervix and a polypectomy was performed. Microscopic findings are consistent with an embryonal rhabdomyosarcoma (botryoide type). A computed tomography of her thorax, abdomen and pelvis were performed and residual disease was found as a mass located at her cervix, which measured approximately 4.5cm in its widest dimensions, without evidence of metastatic disease. Due to the fact that she is young, after discussions in a multidisciplinary meeting, she was subsequently treated with four cycles of multi-agent chemotherapy. Two cycles of chemotherapy and radiotherapy were administered due to the lack of response, but she presented vaginal bleeding with persistence of the same mass in computed tomography. Hence a total interadnexal hysterectomy was made. A histologic examination found residual embryonal rhabdomyosarcoma (botryoide type) located in all her cervix and she is currently under chemotherapy.

**Conclusions:**

The presence of a cervical polyp in an adolescent is a gynecologic oddity and must necessarily be examined histologically because it might be a rhabdomyosarcoma. This is extremely important because diagnosis at an early stage of the disease is a highly favorable prognostic factor that allows “fertility-sparing surgery” for these young patients.

## Introduction

Rhabdomyosarcoma (RMS) is a tumor of skeletal muscle that is classified by the World Health Organization (2013) into embryonal RMS (including botryoide, anaplastic), alveolar RMS (including solid, anaplastic), pleomorphic RMS and spindle cell/sclerosing RMS [1]. RMS is a highly malignant tumor arising from embryonal mesenchyma, and it is the commonest soft tissue sarcoma in childhood and young adults [2]. It accounts for 4 to 6% of all malignancies in this age group [2]. Because of the extreme rarity of cervical RMS, there is a paucity of literature on the subject consisting mainly of case reports [3,4] in which the treatment is not codified [5].

We report a case of embryonal RMS that presented as a cervical polyp protruding from the vaginal introitus in a young teenager, treated by hysterectomy after chemotherapy and radiotherapy. Through this case, we emphasize the diagnosis, management and prognosis factors.

## Case presentation

**Figure 1:**
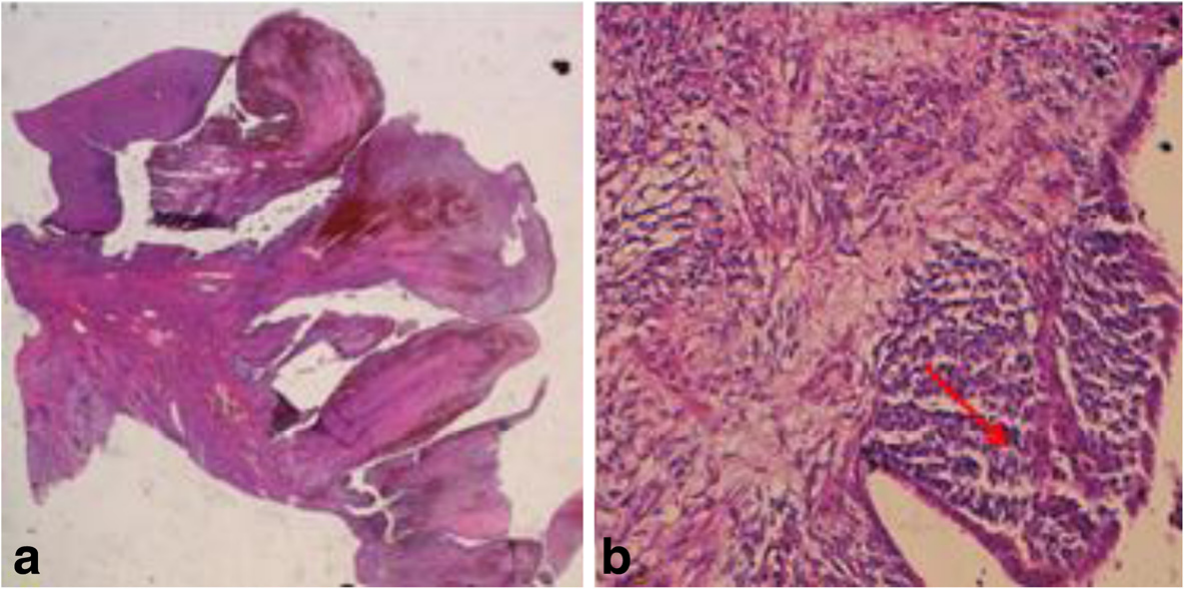
**Sections of tumor showing. a**: Hematoxylin and eosin staining ×40; polypoid formation. **b**: Hematoxylin and eosin staining ×200; the small-rounded tumor cells under the surface epithelium (arrow).

**Figure 2:**
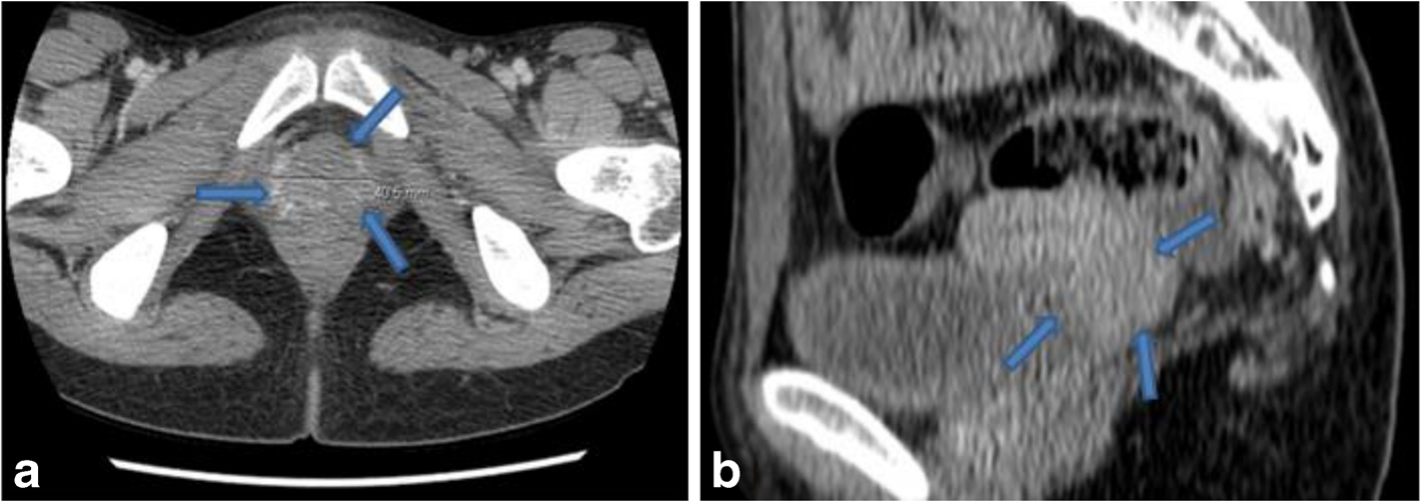
**Pelvic scan with injection of product of contrast. (a)** Axial and **(b)** sagittal section showing a tumor mass in the cervical region (arrows) enhanced by the product of contrast.

A 16-year-old Moroccan girl presented to our center with a protruding mass from her vaginal introitus which she had had for 3 years. It presented as a 6cm polyp with a ‘grape-like’ appearance, smooth, glistening, and focally hemorrhagic. A computed tomography (CT) scan of her pelvis was requested but the family refused due to lack of finance. She was taken to the operating room, at which time the examination revealed a polyp within her vagina, thought to be arising from her cervix and a polypectomy was performed.On microscopic examination, the polyp was covered by squamous and endocervical mucosa. The rhabdomyoblastic nature of the tumor cells was noted by the small-rounded tumor cells, and an eosinophilic cytoplasm with atypical nuclei. Immunohistochemical analysis revealed that tumor cells were immunoreactive to desmin. Nuclear reactivity to myogenin was strong on less than 50% of nuclei. However, the tumor cells were not immunoreactive to cytokeratin. These findings are consistent with an embryonal RMS (botryoide type; Figure 1a, b). A CT of her thorax, abdomen and pelvis were performed, and residual disease was found as a mass located at her cervix, it measured approximately 4.5cm in its widest dimensions (Figure 2a, b), without evidence of metastatic disease.

After discussions in a multidisciplinary meeting (because she is young) she was subsequently treated with four cycles of multi-agent chemotherapy: vincristine, Adriamycin^®^, cyclophosphamide (VAC). Because of a lack of response, two cycles of chemotherapy (cisplatin and etoposide) and radiotherapy were administered, but she presented vaginal bleeding with persistence of the same mass in CT, hence a total interadnexal hysterectomy was made. A histologic examination found residual embryonal RMS located in all her cervix; she is currently under chemotherapy.

## Discussion

When an embryonal RMS affects the genitourinary tract, the vagina is the most common site [6]. Only 0.5% of primary RMSs in girls are found on the cervix [4]; it has been reported in babies as young as 5 months of age [7], but it is usually seen in the second decade of life as is the case in our patient, unlike vaginal lesions which usually present before the age of 4 years [2,4]. The vast majority of RMS cases occur sporadically with no recognized predisposing factor or risk factors, although a small proportion is associated with genetic conditions. Li–Fraumeni cancer susceptibility syndrome, evident by a clustering of soft tissue malignancies (including sarcomas), has been discovered in a family to be caused by a heterozygous germline p.53 mutation [3-8]. Dehner *et al*. also found a link to the pleuropulmonary blastoma family of tumors with confirmed *DICER1* mutations, and reports that RMS in children should be viewed and managed in a broader context to include the possibility of familial pleuropulmonary blastoma tumor predisposition syndrome [3]. Our patient could not seek genetic counseling because of a lack of resources.

Most patients present with a feeling of a mass in the introitus. The tumor, as is the case in our patient, may form soft, grape-like clusters, present as single or multiple polyps [1,4,9]. Additional symptoms included leukorrhea, bleeding, and malodorous discharge [4]. However, the polyp may sometimes take the appearance of a benign mucous polyp relapsed after excision, which may cause a delay in diagnosis [5].

The gross examination showed that the tumors are polypoid with a ‘grape-like’ appearance, smooth, glistening, and focally hemorrhagic [6,10]. They are microscopically characterized by rhabdomyoblasts, and by small round to oval spindled cells [6]. The pathologic differential diagnosis includes benign entities such as rhabdomyoma and an edematous mesodermal cervical polyp (pseudosarcoma botryoides), and malignant entities such as adenosarcoma and other “small, round, blue cell” tumors [1,3].

Primaries RMSs of the cervix are so rare that no single institution has adequate experience to identify superior therapeutic strategies [8-11], but treatment of embryonal RMS of genitourinary primary can be extrapolated to this context. The treatment for sarcoma botryoides of the cervix is traditionally radical, it is a surgery which compromises fertility [11]. However, because the peak incidence of this tumor occurs in young females, patients often desire to retain their fertility potential. The recent literature suggests that sarcoma botryoides of the cervix behave less aggressively than sarcoma botryoides of the vagina and uterus; that is why the management of RMS of the female genital tract has evolved toward conservation of the genitourinary organs [7,5]. Four studies by the Intergroup Rhabdomyosarcoma Study Group (IRSG) [6] reveal that they moved from aggressive surgery (hysterectomy) and radiation to an intensive primary chemotherapy (VAC), without appreciable change in survival (5 years’ survival 82% for surgery/radiation versus 84% for chemotherapy) [7], while maintaining fertility and reduced long-term morbidity from radiation therapy [4,8]. In fact, in addition to VAC chemotherapy, a cone biopsy or a polypectomy are performed to establish a definitive diagnosis, to reduce tumor burden and to minimize the symptomatology of vaginal discomfort, bleeding and vaginal discharge. Radiation is largely reserved for salvage therapy in the recurrent setting for patients of advanced age who could not tolerate intensive chemotherapy [4]. According to the IRSG, fertility-sparing surgery followed by chemotherapy is the appropriate treatment for patients with local disease [11,12] (Table 1). However, there are case reports of unfavorable outcomes despite an adequate surgical excision and chemotherapy, interjecting a note of caution and emphasizing the need for close clinical follow-up [7]. Fertility-sparing surgery should not be considered with the presence of extensive uterine involvement and/or metastasis. The presence of a deep myometrial invasion, a lymphatic invasion and foci of alveolar subtype should prompt discussion with the patient regarding a more aggressive surgical treatment [7].

**Table 1:** **Intergroup Rhabdomyosarcoma Study Group clinical classification system for rhabdomyosarcoma**[4]

**Clinical group, extent of disease, resectability, and margin status**	
I	A: localized tumor confined to site of origin completely resected
	B: localized tumor infiltrating beyond site of origin completely resected
II	A: localized tumor gross total resection but with microscopic residual disease
	B: locally extensive tumor ( spread to regional lymph nodes) completely resected
III	A: localized or locally extensive tumor gross residual disease after biopsy only
	B: localized or locally extensive tumor gross residual disease after major resection (≥50% debulking)
IV	Any size primary tumor with or without regional lymph node involvement with distant metastases irrespective of surgical approach to primary tumor

The embryonal botryoide variant is associated with a much more favorable outcome than the alveolar and the undifferentiated subtypes, which are associated with a particularly poor prognosis [4]. Metastatic disease at presentation and poor response to chemotherapy are strongly associated with poor prognosis [4]. The tendency of genitourinary RMS to spread to regional lymph nodes was reported in 26% of cases and the pelvis was the most common site for primary recurrence (12). The extent of disease following the primary surgical procedure is the most important prognostic factor in these patients [8]. Surgery and chemotherapy are the mainstays of the treatment of cervical RMS, and the prognosis of patients treated with multimodal therapy is very good [4]. In the IRSG, patients with gross residual disease after initial surgery (Clinical Group III) had a 5 years’ survival rate of approximately 70% compared with a greater than 90% 5 years’ survival rate for patients with no residual tumor after surgery (Clinical Group I) and an approximately 80% 5 years’ survival rate for patients with microscopic residual tumor following 7surgery (Clinical Group II) [8].

## Conclusions

The presence of a cervical polyp, particularly one that has achieved a size large enough to protrude from the vagina in a young teenager, is a gynecologic oddity and must necessarily be examined histologically because it might be a RMS. This is extremely important especially as an early disease stage at diagnosis is a highly favorable prognostic factor. Surgery and chemotherapy are the mainstays of treatment of cervical RMS, and the prognosis of patients treated with multimodal therapy is very good.

## Consent

Written informed consent was obtained from the patient and her parents for publication of this case report and any accompanying images. A copy of the written consent is available for review by the Editor-in-Chief of this journal.

## Abbreviations

CT: Computed tomography; IRSR: The Intergroup Rhabdomyosarcoma Study Group; RMS: Rhabdomyosarcoma; VAC: Vincristine, Adriamycin^®^ (doxorubicin), cyclophosphamide.

## Competing interests

The authors declare that they have no competing interests.

## Authors’ contributions

SJ was the principal author and major contributor in writing the manuscript. HB reviewed the literature. LC, IH and AL made the histological study. IK and ST made the radiological study. SA and NM treated the patient in oncology. TB and KH treated the patient in radiotherapy. FZF and HC analyzed and interpreted the data from our patient. MAM corrected the manuscript. All authors read and approved the final manuscript.
